# Persistent cycles and network resilience: a hypernetwork-based framework for temporal graph analysis

**DOI:** 10.1038/s41598-026-44835-4

**Published:** 2026-03-22

**Authors:** Baochen Li, Alfiya Abinova, Shouwei Li

**Affiliations:** 1 School of Aerospace Engineering, Guilin University of Aerospace Technology, Guilin, China; 2 Farabi Business School, Al-Farabi Kazakh National University, Almaty, Kazakhstan; 3 School of Management, Guilin University of Aerospace Technology, Guilin, China

**Keywords:** Temporal networks, Network resilience, Persistent cycles, Temporal cycles, Hypernetwork, Higher-order networks, Temporal efficiency, Robustness, Targeted attacks, Computational biology and bioinformatics, Mathematics and computing, Physics

## Abstract

Temporal networks capture systems whose interactions occur as time-stamped events, where resilience depends on whether time-respecting connectivity can be maintained under disruptions. Existing assessments often rely on static aggregation or path-centric indicators, which may overlook higher-order redundancy that emerges and dissolves over time. We propose a persistence-aware, cycle-driven framework that treats recurrent temporal cycles as resilience-relevant building blocks. The method detects cycles within sliding windows, tracks their recurrence to quantify persistence, and encodes cycles that exceed a persistence threshold as hyperedges in a temporal hypernetwork. Based on this representation, we introduce two dynamic node-level metrics—Temporal Cycle Number (TCN) and Temporal Cycle Ratio (TCR)—to quantify persistent cycle participation and to identify nodes that anchor durable closure. We evaluate the framework on six real-world temporal networks spanning social, transportation, biological, communication, infrastructure, and economic domains using controlled node-removal experiments and temporal-efficiency loss as the primary impact measure. Under the adopted windowing scheme, datasets, and disruption protocols, TCN and TCR exhibit higher rank-based association with disruption impact than the representative static and temporal baselines considered. Moreover, in the same experimental setting, targeted removal of high-TCN/TCR nodes tends to yield larger efficiency degradation than degree-based attacks, which is consistent with the interpretation that recurrent cycle closure can coincide with time-respecting detours that support connectivity. A direct comparison with persistence-weighted scores derived from non-closed temporal motifs (2-paths) further shows that topological closure, rather than motif persistence alone, is the primary driver of the observed predictive advantage. These findings provide empirical support—within the scope of our evaluation—that persistence is an informative factor when using cycle closure as a redundancy signal, and that hypernetwork-encoded persistent cycles offer a compact and interpretable representation for temporal resilience analysis.

## Introduction

Temporal networks model systems whose interactions occur as time-stamped events, including face-to-face contacts, communication logs, transportation schedules, biological activity, and infrastructure operations. A central question in these systems is resilience: how well the network preserves its functional connectivity under disruptions. Conventional resilience assessments often rely on static aggregation or shortest-path indicators^1^, which can obscure time-order constraints and the structural mechanisms that sustain connectivity in evolving networks. In many real systems, connectivity is only meaningful when interactions can be traversed in a time-respecting order; treating all edges as simultaneously available can therefore overstate redundancy and underestimate vulnerability. This gap has become more visible as high-resolution temporal datasets and temporal mining pipelines have proliferated, enabling fine-grained analyses of evolving structures and dynamics. Recent work has shown that temporal motifs can reveal interpretable interaction patterns, but also highlighted caveats when temporal heterogeneity and local counting are not handled carefully^2^.

Cycles provide a natural form of redundancy: when interactions form closed loops, alternative routes remain available after failures, and feedback-like structures can buffer local disruptions. While cycle-based measures have been explored in static settings^1,3^, temporal networks pose a distinct challenge because cycles may emerge, disappear, and re-form as interactions change. Treating the network as a snapshot therefore conflates transient loops with durable ones, potentially misidentifying which structures truly support resilience. The key missing ingredient is *persistence*: if a cycle only appears sporadically, it may not provide dependable alternative routes when disruptions occur. Empirical evidence increasingly suggests that cycle-related motifs capture information beyond degree and pairwise ties. For instance, cycle-motif participation has been linked to measurable differences in individual and collective outcomes in social settings, indicating that closure can encode meaningful structural organization rather than incidental noise^4^. Yet most cycle-based analyses are still performed on aggregated graphs, leaving open the question of which cycles remain repeatedly usable once chronological constraints are enforced.

The resilience objective in this paper is defined on *time-respecting connectivity* and *arrival-time performance*, where disruptions harm the system primarily by eliminating feasible detours ^5^. In this setting, cycles are the *minimal closed redundancy units*: the existence of a cycle is the most direct structural certificate that at least one alternative route can bypass a failed node or edge while preserving time order ^6^. Moreover, cycles admit a compact and interpretable higher-order encoding—a cycle instance can be represented by its node set and naturally mapped to a hyperedge, allowing “closed-loop redundancy groups” to be analyzed without introducing additional semantics ^7,8^. Other temporal motifs are certainly informative ^9,10^, but many (e.g., open triads or 2-paths) do not guarantee a substitute route in the same direct way and are therefore less aligned with the detour-based resilience mechanism studied here. Finally, our framework is *not* cycle-specific in its architecture: it is a generic pipeline of *pattern detection* $\rightarrow$ *persistence tracking* $\rightarrow$ *hyperedge encoding* $\rightarrow$ *node scoring*, with cycles serving as the first concrete and lowest-order closed motif instantiation.

Meanwhile, temporal-network research has advanced representations such as time-varying graphs and higher-order models including hypernetworks^11^. These tools make it possible to preserve time dependence and to encode group-level structures beyond dyadic edges. Higher-order perspectives are particularly relevant in temporal data because interactions often occur in groups or correlated bursts, and higher-order temporal patterns can shape both evolution and predictability^12,13^. Methods that jointly characterize temporal and topological properties of higher-order events further underscore that temporal ordering and structural proximity interact in nontrivial ways, which static summaries may fail to capture^14^. However, a systematic, cycle-centric framework that (i) tracks cycle persistence over time and (ii) connects persistent cycles to resilience outcomes through operational metrics remains underdeveloped. This paper takes a step toward that integration by turning persistent cycles into explicit, measurable higher-order units for temporal resilience analysis. In parallel, advances in temporal community analysis show that temporal structure can switch between stable regimes, motivating representations that track which patterns persist rather than which ones appear briefly^15^.

This paper addresses the gap by proposing a cycle-driven resilience framework for temporal networks. The key idea is to treat *persistent temporal cycles* as higher-order building blocks: cycles that repeatedly appear across time windows are encoded as hyperedges in a temporal hypernetwork, and their node-level participation is summarized by dynamic metrics that can be used to anticipate vulnerability under targeted disruptions. Our aim is not to count cycles exhaustively, but to identify *recurrently available* closure that can support time-respecting detours when failures occur.

To clarify the modeling scope, we represent each dataset as a sequence of windowed temporal snapshots derived from time-stamped interactions. Within each analysis window, the network is treated as a simple temporal graph $G_t=(V,E_t)$ with a fixed node set $V$ over the observation horizon; if a node is inactive in a given window, it remains in $V$ but has no incident edges in that snapshot. Repeated interactions between the same pair of nodes within a window are aggregated into a single edge for the main analysis, so that cycle detection is performed on unweighted simple snapshots unless edge weights are explicitly introduced in n comparisons. Unless otherwise stated, the main framework uses an undirected representation within each window, which provides a unified basis for identifying recurrent closure and comparing resilience patterns across heterogeneous datasets.

Our contributions are fourfold. First, we present a temporal cycle detection and tracking procedure that distinguishes transient loops from cycles with sustained duration. Second, we develop a temporal hypernetwork representation that encodes persistent cycles as time-evolving hyperedges, enabling compact higher-order analysis. Third, we introduce two dynamic cycle-based resilience metrics—Temporal Cycle Number (TCN) and Temporal Cycle Ratio (TCR)—and evaluate them on six real-world temporal networks using controlled disruption experiments and association analysis with temporal-efficiency loss. Fourth, to empirically assess whether the focus on cycles is justified beyond conceptual arguments, we perform a motif-substituted comparison under the same persistence-aware pipeline, replacing the cycle detector with detectors for persistent 2-paths on representative datasets. This evaluation directly links structure to function by testing whether persistence-aware cycle participation improves the ability to anticipate temporal-efficiency degradation under node removals, rather than reporting descriptive cycle statistics alone.

The results show that persistent cycles behave as resilience backbones: they provide topological redundancy that stabilizes temporal connectivity under perturbations, and nodes strongly embedded in persistent cycles act as “cycle anchors” whose removal yields disproportionate efficiency degradation. These findings complement path-based and snapshot-based resilience indicators by highlighting higher-order temporal structures that are otherwise easy to miss.

The remainder of this paper is organized as follows. “Problem definition and preliminaries” defines the temporal network setting, time-respecting connectivity, and temporal cycle persistence. “Method: persistent-cycle hypernetwork framework” presents the persistent-cycle hypernetwork framework and the TCN/TCR metrics. “Data and experimental design” describes datasets, baselines, and the disruption-based evaluation design. “Results” reports empirical results across six temporal networks. “Discussion” discusses implications, limitations, and future directions, and “Conclusion” concludes.

## Problem definition and preliminaries

### Temporal network model and windowing

Temporal networks represent interactions as time-stamped edges, enabling analysis of time-dependent reachability and evolving connectivity^16^. Let a temporal network be observed over an interval $[0,T]$ with a fixed node set $V$ and a collection of time-stamped interactions. For empirical analysis, we discretize time into consecutive (possibly overlapping) windows and derive a sequence of snapshots $\{G_t=(V,E_t)\}_{t=1}^{T_w}$, where $E_t$ contains interactions whose timestamps fall into window $t$. Sliding windows allow tracking how structures emerge, persist, and dissolve over consecutive intervals^17^. Unless otherwise stated, each windowed snapshot $G_t=(V,E_t)$ is treated as an undirected, unweighted graph in the main analysis.

Windowing involves a trade-off between temporal resolution and structural stability. Smaller windows preserve ordering but may be sparse and yield few detectable cycles, whereas larger windows increase density but risk mixing interactions that are not jointly feasible in time. This paper uses windowed snapshots as the operational substrate for detecting cycles, tracking their recurrence, and quantifying persistence.

### Time-respecting connectivity and temporal efficiency

In temporal systems, redundancy is only useful when alternative routes are feasible under time ordering. A *time-respecting path* (or temporal path) is a sequence of edges whose timestamps are non-decreasing, so that the path can be traversed without violating causality^18^. This constraint is central to resilience assessment: disruptions matter because they can break not only topological connections but also the availability of time-ordered routes.

To quantify global connectivity loss under disruptions, we rely on temporal efficiency, a widely used measure that summarizes how quickly nodes can reach one another through time-respecting paths^19^. At a high level, temporal efficiency decreases when disruptions lengthen earliest-arrival times or make some node pairs temporally unreachable. In later sections, we measure resilience impact as the relative drop in temporal efficiency after node removals, denoted by $\Delta E$ (defined explicitly in “Evaluation metrics and statistical analysis”).

### Temporal cycles and persistence

Cycles are fundamental redundancy structures: a cycle provides an alternative route between nodes when some links fail. In static graphs, a cycle is a closed path with no repeated vertices except the start/end node^20^. In directed graphs, cycles may correspond to feedback mechanisms, whereas in undirected graphs they represent structural alternatives and local connectivity reinforcement^21^. In the present study, the operational definition follows the undirected case described above. Cycle enumeration and analysis have been studied extensively for static graphs^22^.

Temporal networks require an additional constraint: a cycle should be feasible under chronological order. Temporal cycles therefore differ from static cycles because they must satisfy both topological closure and time consistency^23^. Intuitively, a temporal cycle is meaningful for resilience only if its constituent interactions can be traversed in a time-respecting sequence within (or across) analysis windows.

This paper focuses on *persistence* as the key property that separates reliable redundancy from incidental closure. A snapshot-based cycle count implicitly assumes all edges coexist simultaneously, which can be misleading when interactions are time-stamped and not jointly available in real time. By tracking whether a given cycle reappears across successive windows, we distinguish transient loops from cycles that repeatedly provide time-respecting alternatives. These persistent cycles form the building blocks of our method: they are encoded as hyperedges in a temporal hypernetwork and summarized by node-level dynamic metrics in “Method: persistent-cycle hypernetwork framework”.

## Method: persistent-cycle hypernetwork framework

This section presents our persistent-cycle hypernetwork framework, which turns recurring temporal cycles into explicit higher-order units and derives node-level metrics for resilience analysis. The workflow consists of (i) detecting cycles within each temporal window, (ii) tracking cycle recurrence across windows to quantify persistence, (iii) encoding persistent cycles as hyperedges in a temporal hypernetwork, and (iv) computing dynamic node-level metrics (TCN/TCR) used for disruption-based validation.

### Cycle detection in temporal windows

Given windowed snapshots $\{G_t=(V,E_t)\}$ defined in “Temporal network model and windowing”, we first identify candidate cycles within each snapshot. At this stage, we treat a cycle as a closed walk in the snapshot graph, and defer the persistence decision to the tracking step. This separation is deliberate: it prevents a single dense window from dominating the analysis and allows persistence to be defined across windows rather than within one snapshot.

### Cycle matching and persistence tracking

For a temporal network $G(t) = (V(t), E(t))$ observed over discrete time steps $t \in \{1,\ldots ,T\}$, we define a persistence indicator for cycle $c$:

1 $$\begin{aligned} \phi (c,t) = {\left\{ \begin{array}{ll} 1 & \text {if cycle } c \text { exists at time } t \\ 0 & \text {otherwise} \end{array}\right. } \end{aligned}$$

In this work, the statement “cycle *c* exists at time *t*” is evaluated on the windowed snapshot $G_t=(V,E_t)$. Formally, we use the operational definition

2 $$\begin{aligned} \phi (c,t)=1 \;\Longleftrightarrow \; c \subseteq G_t, \end{aligned}$$

i.e., all edges required by cycle *c* are present in the snapshot induced by window *t*. This window-based definition matches the cycle-detection step in “Cycle detection in temporal windows” and ensures that persistence is measured across windows rather than assumed from a static aggregation.

The temporal duration of cycle $c$ is:

3 $$\begin{aligned} \tau (c) = \sum _{t=1}^T \phi (c,t) \cdot \Delta t \end{aligned}$$

where $\Delta t$ is the duration of one time step (or one analysis window). For each dataset, $\Delta t$ is fixed throughout the observation period, so $\tau (c)$ is monotonically equivalent to the number of windows in which cycle $c$ appears. Accordingly, cycles that reappear more often receive larger $\tau (c)$, while one-off loops remain low-weight. Because window durations differ across datasets, the absolute value of $\tau (c)$ is interpreted only within a dataset and is not used for direct cross-dataset magnitude comparisons.

Operationally, we maintain a cycle inventory over the sliding windows. At each time step $t$, the procedure (i) detects cycles in the current window, (ii) matches them to previously observed cycles (to update $\tau (c)$), and (iii) records newly formed and dissolved cycles. Cycle matching is performed on the cycle node set (or an equivalent canonical representation) so that the same cycle can be tracked even when it disappears and later reappears. This tracking perspective captures recurrence patterns that are typically invisible in static aggregation.

### Persistent-cycle hypernetwork construction

To represent persistent higher-order redundancy compactly, we construct a temporal hypernetwork $H = (V, \mathcal {E})$, where nodes $V$ are inherited from the original temporal network and hyperedges $\mathcal {E}$ encode cycles whose duration exceeds a threshold $\tau _{min}$:

4 $$\begin{aligned} e_c = \{v \in V \mid v \in c \text { and } \tau (c) \ge \tau _{min}\} \end{aligned}$$

The threshold $\tau _{min}$ is a modeling choice that filters out transient closure and retains cycles that are repeatedly available as potential alternative routes. For notational clarity, each retained hyperedge $e_c$ is indexed by its generating cycle $c$, so that the mapping $c \mapsto e_c$ is one-to-one for the persistent cycles included in $\mathcal {E}$. We treat $\tau _{min}$ as a tunable parameter and determine it in the experimental design (“Evaluation metrics and statistical analysis”) to balance informativeness and stability.

The hypernetwork adjacency $A_H$ summarizes co-participation in persistent cycles:

5 $$\begin{aligned} A_H(i,j) = \sum _{e \in \mathcal {E}} \mathbb {I}_{\{v_i,v_j \in e\}} \cdot w(e) \end{aligned}$$

where $\mathbb {I}$ is an indicator function and the hyperedge weight $w(e)$ is defined deterministically in our main analysis. Specifically, for each persistent-cycle hyperedge $e_c \in \mathcal {E}$ induced by cycle $c$ (Eq. 5), we set

6 $$\begin{aligned} w(e_c) = \tau (c). \end{aligned}$$

That is, the hypernetwork adjacency $A_H(i,j)$ accumulates the persistence durations of all retained cycles in which nodes $i$ and $j$ co-participate. Alternative weighting schemes (e.g., length-normalized weights) are possible, but are not used in the main experiments to keep the framework closed and reproducible. Intuitively, $A_H(i,j)$ increases when $i$ and $j$ repeatedly co-occur in durable cycles, capturing a higher-order redundancy relation that is not visible from dyadic edges alone.

**Fig. 1:**
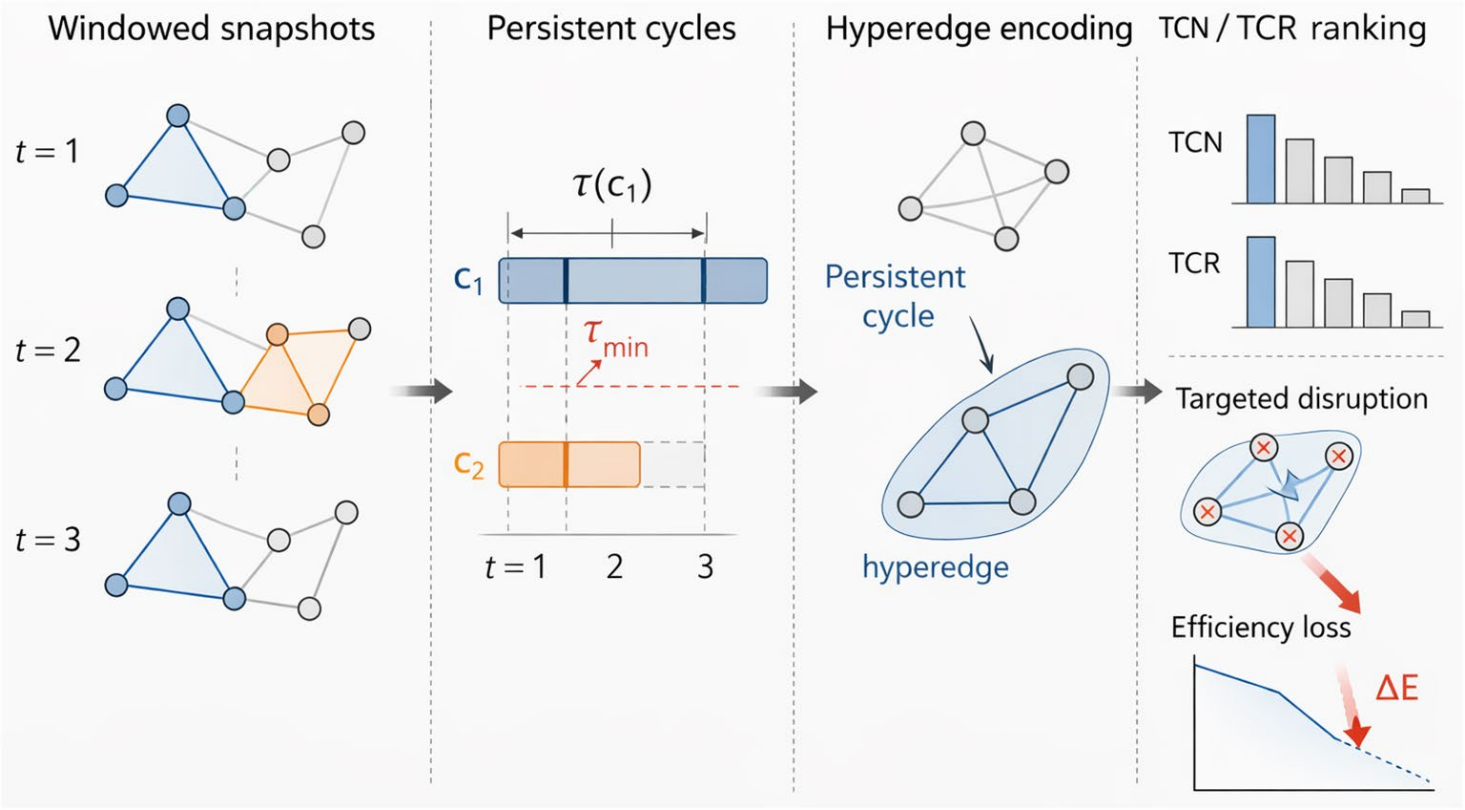
Conceptual illustration of the proposed persistence-aware cycle-driven framework. Windowed temporal snapshots are used to identify recurrent cycles, whose persistence is tracked across time and filtered by a threshold. The retained cycles are encoded as hyperedges in a higher-order representation, from which TCN/TCR-based node rankings are derived and evaluated through targeted disruption and temporal-efficiency loss..

As illustrated in Fig. 1, the proposed pipeline extends standard temporal analysis modules by inserting a cycle-driven branch: temporal cycle detection/tracking yields persistent cycles, which are then encoded as hyperedges and summarized by dynamic metrics. These metrics are designed to be directly testable under disruptions, linking higher-order temporal structure to resilience outcomes.

### Dynamic cycle-based metrics: TCN and TCR

Building on cycle tracking and hypernetwork encoding, we define two node-level metrics.

Temporal Cycle Number (TCN) for node $i$ over window $[t_1,t_2]$ quantifies weighted participation in cycles that occur within the window:

7 $$\begin{aligned} TCN_i(t_1,t_2) = \sum _{c \in C_i(t_1,t_2)} \tau (c) \cdot s(c,i) \end{aligned}$$

where $C_i(t_1,t_2)$ is the set of cycles containing node $i$ in the window, $\tau (c)$ is the cycle duration (or persistence weight), and $s(c,i)$ captures node $i$’s structural role within cycle $c$ (e.g., a cycle-specific importance score). To keep TCN/TCR fully interpretable and to avoid introducing an additional tunable component, we set $s(c,i)=1$ for all (*c*, *i*) in all main experiments, so that TCN reduces to a persistence-weighted count of cycles containing node *i*. More elaborate role weights (e.g., length-regularized contributions or temporally feasible traversal roles) can be incorporated as an extension, but are not required for the core results reported here. TCN increases when a node participates in many cycles that are repeatedly present across windows, reflecting durable redundancy involvement rather than momentary closure.

Temporal Cycle Ratio (TCR) normalizes cycle participation by the node’s temporal connectivity:

8 $$\begin{aligned} TCR_i(t_1,t_2) = \frac{TCN_i(t_1,t_2)}{\sum _{t=t_1}^{t_2} k_i(t) + \epsilon } \end{aligned}$$

where $k_i(t)$ is node $i$’s degree at time $t$ and $\epsilon$ avoids division by zero. By controlling for activity level, TCR highlights nodes whose cycle-based redundancy is high relative to how frequently they interact. The denominator represents cumulative temporal exposure to dyadic interactions over the observation window. By dividing TCN by this exposure term, TCR estimates how efficiently a node’s interaction activity is converted into persistent higher-order closure. This normalization helps distinguish nodes that are simply highly active from nodes whose interactions repeatedly participate in durable cyclic redundancy.

In the disruption experiments (“Data and experimental design”), nodes with high TCN/TCR behave as “cycle anchors”: removing them eliminates durable alternative routes and produces disproportionate temporal-efficiency loss.

### Toy example: step-by-step computation of persistence, TCN, and TCR

**Fig. 2:**
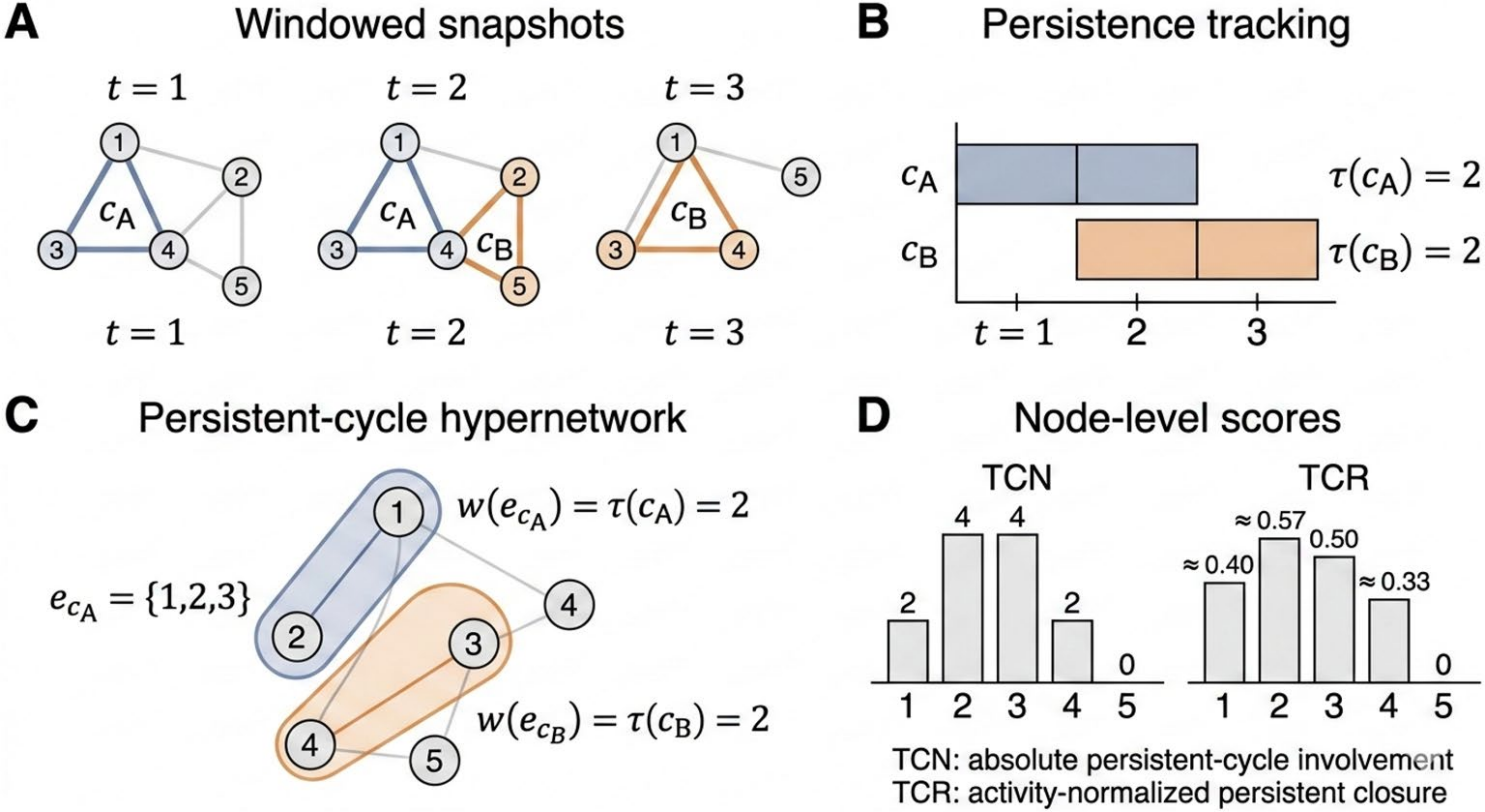
Visual toy example of persistent-cycle tracking and TCN/TCR computation.

We provide a small hand-calculable example to illustrate (i) window-based cycle detection, (ii) cycle matching and persistence accumulation, and (iii) the resulting node-level scores TCN and TCR. Figure 2 provides a visual summary of this toy example, showing how recurrent cycles are identified across windows, accumulated into persistence values, encoded as hyperedges, and finally translated into node-level TCN/TCR scores.

Step1: Setup. Consider a temporal network on five nodes $V=\{1,2,3,4,5\}$ observed over three equal-length windows ($T=3$) with duration $\Delta t=1$. For each window *t*, we build the snapshot $G_t=(V,E_t)$ and detect simple cycles in $G_t$.

Step2: Windowed snapshots and detected cycles. Table 1 lists the snapshot edges and the cycles detected in each window. For simplicity, we focus on two 3-cycles (triangles), which are the smallest non-trivial cycles in undirected graphs. As illustrated in Panel A of Fig. 2, cycle $c_A$ appears in the first two windows, while cycle $c_B$ appears in the last two windows.

**Table 1:** Toy example: windowed snapshots and detected cycles.

Window *t*	Edges $E_t$	Detected cycles in $G_t$ (node sets)
$t=1$	$\{(1,2),(2,3),(3,1),(3,4),(4,5)\}$	$c_A=\{1,2,3\}$
$t=2$	$\{(1,2),(2,3),(3,1),(2,4),(3,4),(2,5)\}$	$c_A=\{1,2,3\},\; c_B=\{2,3,4\}$
$t=3$	$\{(2,3),(3,4),(4,2),(1,5)\}$	$c_B=\{2,3,4\}$

Step3: Cycle matching and persistence accumulation. We match cycles across windows using a canonical representation of the node set. Thus, the triangle on $\{1,2,3\}$ is treated as the same cycle $c_A$ whenever it appears, and similarly for $c_B=\{2,3,4\}$. Using the window-based existence definition $\phi (c,t)=1 \Longleftrightarrow c\subseteq G_t$, we obtain:

$$\phi (c_A,1)=1,\;\phi (c_A,2)=1,\;\phi (c_A,3)=0;\quad \phi (c_B,1)=0,\;\phi (c_B,2)=1,\;\phi (c_B,3)=1.$$

Therefore, by Eq. (3) with $\Delta t=1$,

$$\tau (c_A)=\sum _{t=1}^3 \phi (c_A,t)\Delta t = 2,\qquad \tau (c_B)=\sum _{t=1}^3 \phi (c_B,t)\Delta t = 2.$$

This recurrence pattern is visualized in Panel B of Fig. 2, where the two cycles have identical persistence duration even though they are active in different windows.

Step4: Node-level TCN (with the paper’s default $s(c,i)=1$). We compute $TCN_i$ over the full interval $[t_1,t_2]=[1,3]$ using Eq. (7) with $s(c,i)=1$. Nodes in $c_A$ are $\{1,2,3\}$ and nodes in $c_B$ are $\{2,3,4\}$, so:

$$TCN_1 = \tau (c_A)=2,\quad TCN_2 = \tau (c_A)+\tau (c_B)=4,\quad$$

$$TCN_3 = \tau (c_A)+\tau (c_B)=4,\quad TCN_4 = \tau (c_B)=2,\quad TCN_5 = 0.$$

Panel C of Fig. 2 further shows how the two persistent cycles are encoded as weighted hyperedges, which makes the transition from cycle persistence to node-level scoring visually explicit.

Step5: Node activity and TCR. Next, we compute the windowed degrees $k_i(t)$ in each snapshot $G_t$ and sum them over $t=1,2,3$ (Eq. (8)). From Table 1, the results are showed in Table  2. The resulting TCN and TCR values are summarized visually in Panel D of Fig. 2, where nodes 2 and 3 have the largest TCN, while node 2 attains the highest TCR after normalization by cumulative activity.

**Table 2:** Toy example: degrees and TCR computation over $t=1,2,3$.

Node *i*	$k_i(1)$	$k_i(2)$	$k_i(3)$	$\sum _{t=1}^3 k_i(t)$	$TCR_i=\frac{TCN_i}{\sum k_i(t)+\epsilon }$
1	2	2	1	5	$\approx 2/5=0.40$
2	1	4	2	7	$\approx 4/7=0.57$
3	3	3	2	8	$=4/8=0.50$
4	2	2	2	6	$\approx 2/6=0.33$
5	1	1	1	3	$=0$

In this toy example, nodes 2 and 3 obtain the largest TCN because they participate in both persistent cycles, while node 2 has the highest TCR because its cycle persistence is high relative to its cumulative activity. This demonstrates the intended distinction: TCN captures absolute persistent-cycle involvement, whereas TCR highlights nodes whose persistent-cycle redundancy is high after controlling for temporal activity.

### Computational complexity and implementation details

The computational cost of the framework is dominated by cycle detection and tracking. Cycle enumeration can be expensive in dense graphs because the number of cycles may grow rapidly with network size; in practice, windowing reduces snapshot density and makes the problem tractable for the datasets considered here. Tracking adds overhead proportional to the number of detected cycles per window, since each cycle must be mapped to a canonical representation and updated in the inventory.

Hypernetwork construction is linear in the number of retained persistent cycles after applying $\tau _{min}$, and metric computation (TCN/TCR) is linear in the number of (node, cycle) incidences among those retained cycles. To ensure reproducibility, our experiments use a fixed windowing scheme per dataset and compute all rankings on the pre-disruption network without re-ranking after removals (details in “Data and experimental design”).

## Data and experimental design

This section describes the datasets, preprocessing choices, baselines, and disruption-based evaluation protocol used to test whether persistent cycle participation predicts resilience loss. All methods are evaluated under matched windowing settings per dataset and identical disruption budgets to ensure comparability.

### Datasets

We selected six *publicly available* temporal network datasets spanning distinct domains to ensure broad applicability. For transparency and reproducibility, we provide the *primary source* and a *direct access link* for each dataset.

1. Social interactions: face-to-face proximity events recorded in a scientific conference setting, using the *Hypertext 2009 dynamic contact network* released by the SocioPatterns project (https://sociopatterns.org/datasets/hypertext-2009-dynamic-contact-network/)^24,25^. Nodes represent individuals and time-stamped edges indicate close-range contacts.
2. Transportation systems: air–traffic interaction networks constructed from *OpenSky Network* ADS-B records (https://opensky-network.org/)^26,27^, where nodes are airports and time-stamped edges are derived from observed flight movements between airports.
3. Biological networks: neural activity/connectivity time series from the *hc-3* dataset hosted by CRCNS.org (https://crcns.org/data-sets/hc/hc-3; DOI: 10.6080/K09G5JRZ)^28,29^, contributed by the Buzsáki Lab, where temporal events are derived from recorded spike/LFP data.
4. Communication networks: email exchange records using the *Enron Email Dataset* maintained by Carnegie Mellon University (https://www.cs.cmu.edu/~./enron/)^30,31^, where nodes are users and time-stamped edges represent email interactions.
5. Infrastructure networks: power-grid topology from the *Western States Power Grid* dataset (http://www-personal.umich.edu/~mejn/netdata/)^32,33^; temporal edge-failure sequences are generated/used in the spirit of cascade studies on this benchmark topology.
6. Economic networks: international trade networks built from official *UN Comtrade* monthly/annual trade statistics (https://comtrade.un.org/) and the UN Comtrade API portal (https://comtradedeveloper.un.org/)^34–36^, aggregated into time-indexed country-to-country trade links.

Access notes: some providers (e.g., OpenSky and CRCNS) may require free registration and/or data-access requests; all links above point to the original provider portals used to obtain the data. These datasets differ in temporal granularity and in the operational meaning of edges, providing a stress test for the proposed metrics across heterogeneous dynamics.

### Preprocessing and parameter settings

Each dataset was preprocessed to ensure temporal consistency, with edge timestamps aligned to a common reference frame. We treat networks as simple temporal graphs within each analysis window , and unless otherwise stated, each windowed snapshot is represented as an undirected, unweighted graph. Multi-edges within a window are aggregated as a single interaction. For datasets whose raw records are naturally directed (e.g., email exchanges or trade flows), directed interactions are symmetrized within each window for the main analysis so that cycle detection and resilience comparisons are conducted under a common representation.

We applied sliding time windows of duration $\Delta t$ matched to each dataset’s characteristic timescale, ranging from minutes (social networks) to months (trade networks). Window size and step were chosen to balance temporal resolution and structural stability: smaller windows capture fast dynamics but may yield sparse snapshots with few cycles, whereas larger windows increase cycle density but risk mixing incompatible interaction phases. To make results interpretable and comparable within each domain, we choose $\Delta t$ so that snapshots are neither dominated by isolated edges nor nearly fully aggregated. For networks with continuous-time edge activations, we employed exponential kernels to smooth temporal interactions^37^.

To reduce sensitivity to a single window choice, we report results under the primary $\Delta t$ for each dataset and verify that the main conclusions remain unchanged under moderate perturbations of $\Delta t$ (“Discussion”).

Because $\Delta t$ differs across datasets, persistence values are interpreted within-dataset; cross-dataset comparisons rely on rank-based statistics rather than absolute persistence magnitudes.

Because cycle detection and persistence estimation depend on windowing, we explicitly evaluate the stability of TCN/TCR rankings under systematic variations of the window size. Let $\Delta t_0$ denote the primary window size used in the main experiments for each dataset. We consider a multiplicative set $\Delta t \in \{0.5\Delta t_0,\,0.75\Delta t_0,\,\Delta t_0,\,1.5\Delta t_0,\,2\Delta t_0\}$, and use a fixed overlap ratio (50% overlap, i.e., step size $=0.5\Delta t$) to keep temporal coverage comparable across window sizes. For each $\Delta t$, we recompute cycle inventories, persistence $\tau (c)$, and node rankings of TCN/TCR on the same observation horizon. Ranking consistency is quantified against the baseline window $\Delta t_0$ using Spearman’s $\rho$ (and Kendall’s $\tau$ as a robustness check), reported in “Sensitivity to window size and ranking stability”.

### Baseline methods

We compared our cycle-based resilience metrics against three categories of established approaches:

1. Static network metrics: traditional indicators including betweenness centrality^38^ and k-core decomposition^39^, computed on time-aggregated snapshots.
2. Temporal extensions: dynamic versions of centrality measures for temporal networks, such as temporal betweenness^40^ and temporal eigenvector centrality^41^.
3. Path-based methods: connectivity metrics based on temporal paths^18^, including reachability and temporal efficiency measures.
4. Alternative temporal motif baselines: node-level scores derived from *persistent 2-paths* (open triads) ^9,10^, computed through the identical persistence-tracking pipeline as cycles but with a different motif detector *M*, enabling a controlled comparison of motif type rather than framework architecture.

All baseline implementations used publicly available code repositories with parameters set according to original publications. When multiple parameterizations were available (e.g., time-decay factors), we selected the recommended default settings and kept them fixed across datasets to avoid overfitting baselines. When a baseline depends on windowing (e.g., temporal centralities), we compute it on the same snapshot sequence used by our method.

### Disruption protocols

The evaluation consists of controlled disruption experiments and, where recorded disruptions are available, event-aligned checks:

1. Controlled disruption tests: we simulate resilience by removing nodes ranked by each metric (including TCN/TCR) and measuring connectivity degradation. The removal strategies include:
  Targeted removal of top-ranked nodes
  Random failures
  Hybrid strategies combining both approaches
  Unless otherwise stated, rankings are computed on the pre-disruption temporal network and removals are executed without re-ranking, which isolates the predictive value of each metric as a vulnerability indicator.
2. Real-event alignment: for datasets with recorded disruption events (e.g., infrastructure failures), we align metric trajectories with event windows and examine associations with observed impact patterns.

We evaluate multiple removal budgets to capture both early-stage fragility (small fractions removed) and systemic breakdown (larger fractions removed).

### Evaluation metrics and statistical analysis

The primary impact measure is the relative drop in global temporal efficiency:

9 $$\begin{aligned} \Delta E = \frac{E_{initial} - E_{disrupted}}{E_{initial}} \end{aligned}$$

where $E$ is temporal efficiency^19^.

Let $\mathcal {R}$ denote the set of ordered node pairs (*u*, *v*) for which a time-respecting path exists within the observation horizon under the adopted windowing scheme, and let $|\mathcal {R}|$ be its cardinality. We define the temporal reachability ratio as

10 $$\begin{aligned} \textrm{TRR}=\frac{|\mathcal {R}|}{|V|(|V|-1)} , \end{aligned}$$

and report reachability loss under disruption as

11 $$\begin{aligned} \Delta \textrm{TRR}=\frac{\textrm{TRR}_{initial}-\textrm{TRR}_{disrupted}}{\textrm{TRR}_{initial}} . \end{aligned}$$

This metric complements $\Delta E$ by separating “unreachable” failures from mere delays in arrival times.

For each disrupted temporal network, we compute the size of the largest temporal (weakly) connected component under time-respecting reachability, denoted by $\textrm{LTCC}$, and normalize it as $\textrm{LTCC}/|V|$. We report its relative degradation as

12 $$\begin{aligned} \Delta \textrm{LTCC}=\frac{\textrm{LTCC}_{initial}-\textrm{LTCC}_{disrupted}}{\textrm{LTCC}_{initial}} . \end{aligned}$$

This quantifies fragmentation effects that may not be fully reflected by efficiency changes alone.

For statistical validation, each stochastic experiment was repeated with 100 different random seeds. We summarize results using mean ± standard deviation across runs and use paired tests when comparing strategies under identical seeds. Rank-based associations between node scores and observed $\Delta E$ are reported using correlation on ranked values to reduce sensitivity to scaling differences across metrics and datasets. This rank-based evaluation also avoids conflating cross-dataset differences in $\Delta t$ with differences in the substantive persistence structure captured by the proposed metrics.

The persistence threshold $\tau _{min}$ is selected to balance (i) ranking reproducibility and (ii) sufficient retained cycle instances. Concretely, for each dataset we construct a candidate set $\mathcal {T}=\{Q_{50},Q_{60},Q_{70},Q_{80},Q_{90}\}$ where $Q_{p}$ denotes the *p*-th percentile of observed cycle durations $\tau (c)$ under the baseline window $\Delta t_0$. For each candidate $\tau _{min}\in \mathcal {T}$, we compute TCN/TCR rankings across consecutive windows and measure the average adjacent-window rank consistency using Spearman’s $\rho$. We choose the $\tau _{min}$ that maximizes this stability score subject to a minimum retained-cycle constraint (at least $\eta$ persistent cycles; we set $\eta$ to avoid degenerate cases where hyperedges become too sparse for meaningful comparison).

## Results

This section evaluates whether persistent cycle participation provides predictive signals for vulnerability under disruptions. We report (i) rank-based associations between metric scores and observed efficiency loss, (ii) node-level stratification patterns, (iii) temporal evolution of cycle participation, (iv) comparative damage under different disruption strategies, and (v) hypernetwork-level observations enabled by the persistent-cycle encoding.

### Predictive performance of resilience metrics

We first assess how well each metric anticipates disruption impacts. Table 3 reports the association between node scores and observed efficiency loss $\Delta E$ under targeted removals. We use rank correlation to reduce sensitivity to scale differences across metrics and datasets.

**Table 3:** Predictive performance of resilience metrics.

Metric	Social	Transport	Biological	Communication	Infrastructure	Economic
TCN	0.87	0.82	0.91	0.85	0.88	0.83
TCR	0.85	0.79	0.89	0.82	0.86	0.81
T-Bet	0.72	0.68	0.75	0.71	0.69	0.70
T-Eff	0.65	0.63	0.68	0.64	0.62	0.66
S-Bet	0.58	0.55	0.61	0.57	0.53	0.59

*TCN* temporal cycle number, *TCR* temporal cycle ratio, *T-Bet* temporal betweenness, *T-Eff* temporal efficiency, *S-Bet* static betweenness.

The cycle-based metrics yield higher rank-based associations than the listed baselines across the six datasets *under the evaluation setting used in this paper* (i.e., the selected windowing parameters, persistence thresholding rule, and node-removal protocol). The performance gap is particularly pronounced in the transportation and communication networks in our experiments, where strict time ordering can limit feasible temporal alternatives. We emphasize that these values quantify association under the specified disruption protocol rather than causal necessity; absolute magnitudes may vary with removal budgets, window choices, preprocessing decisions (e.g., symmetrization of directed interactions), and baseline parameterizations.

### Sensitivity to window size and ranking stability

**Fig. 3:**
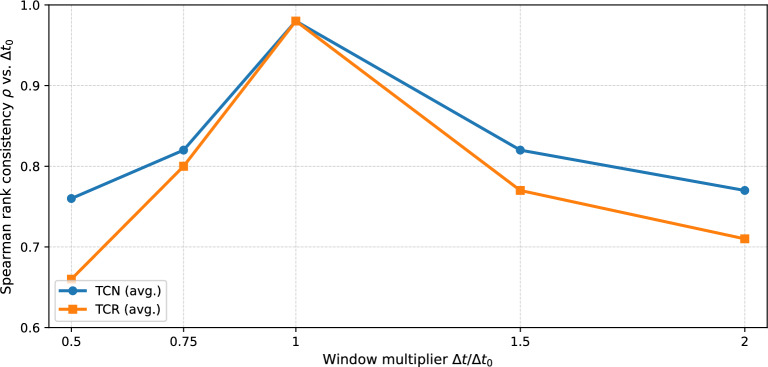
Ranking stability of TCN/TCR under window-size variations. Values show Spearman’s $\rho$ between rankings obtained with window $\Delta t$ and the baseline $\Delta t_0$, averaged over representative datasets (social/transport/economic).

Because window size affects snapshot density, cycle detectability, and persistence accumulation, we evaluate whether TCN/TCR rankings remain stable under systematic window-size changes. For each representative dataset (social/transport/economic), we compute TCN and TCR rankings under $\Delta t \in \{0.5\Delta t_0,0.75\Delta t_0,\Delta t_0,1.5\Delta t_0,2\Delta t_0\}$ and quantify rank consistency with the baseline $\Delta t_0$ using Spearman’s $\rho$. Figure 3 summarizes ranking stability as a function of the window multiplier, and Table 4 reports dataset-level values. Overall, TCN/TCR rankings are most stable near $\Delta t_0$ (0.75–1.5$\times$), and degrade gracefully only at extreme window sizes where snapshots become either too sparse (0.5$\times$) or overly aggregated (2$\times$) (Table 5).

**Table 4:** Dataset-level ranking stability under window-size variations (Spearman’s $\rho$ vs. $\Delta t_0$).

	TCN: $\rho (\textrm{rank}_{\Delta t},\textrm{rank}_{\Delta t_0})$					TCR: $\rho (\textrm{rank}_{\Delta t},\textrm{rank}_{\Delta t_0})$				
Dataset	0.5$\times$	0.75$\times$	1$\times$	1.5$\times$	2$\times$	0.5$\times$	0.75$\times$	1$\times$	1.5$\times$	2$\times$
Social	0.71	0.76	1.00	0.77	0.69	0.67	0.79	1.00	0.72	0.59
Transport	0.74	0.77	1.00	0.72	0.70	0.59	0.70	1.00	0.66	0.65
Economic	0.80	0.81	1.00	0.81	0.81	0.58	0.78	1.00	0.73	0.71

**Table 5:** Performance stability under window-size variations: average improvement of TCN/TCR over the best baseline on rank-association with $\Delta E$.

Metric	0.5$\times$	0.75$\times$	1$\times$	1.5$\times$	2$\times$
TCN: $\Delta \rho$ vs best baseline	+0.15	+0.20	+0.18	+0.19	+0.14
TCR: $\Delta \rho$ vs best baseline	+0.10	+0.18	+0.14	+0.17	+0.09

$\Delta \rho$ denotes the increase in rank-based association (Spearman) with disruption impact $\Delta E$ relative to the strongest baseline among {T-Bet, T-Eff, S-Bet, etc.} under the same $\Delta t$.

### Node-level stratification and cycle anchors

**Fig. 4:**
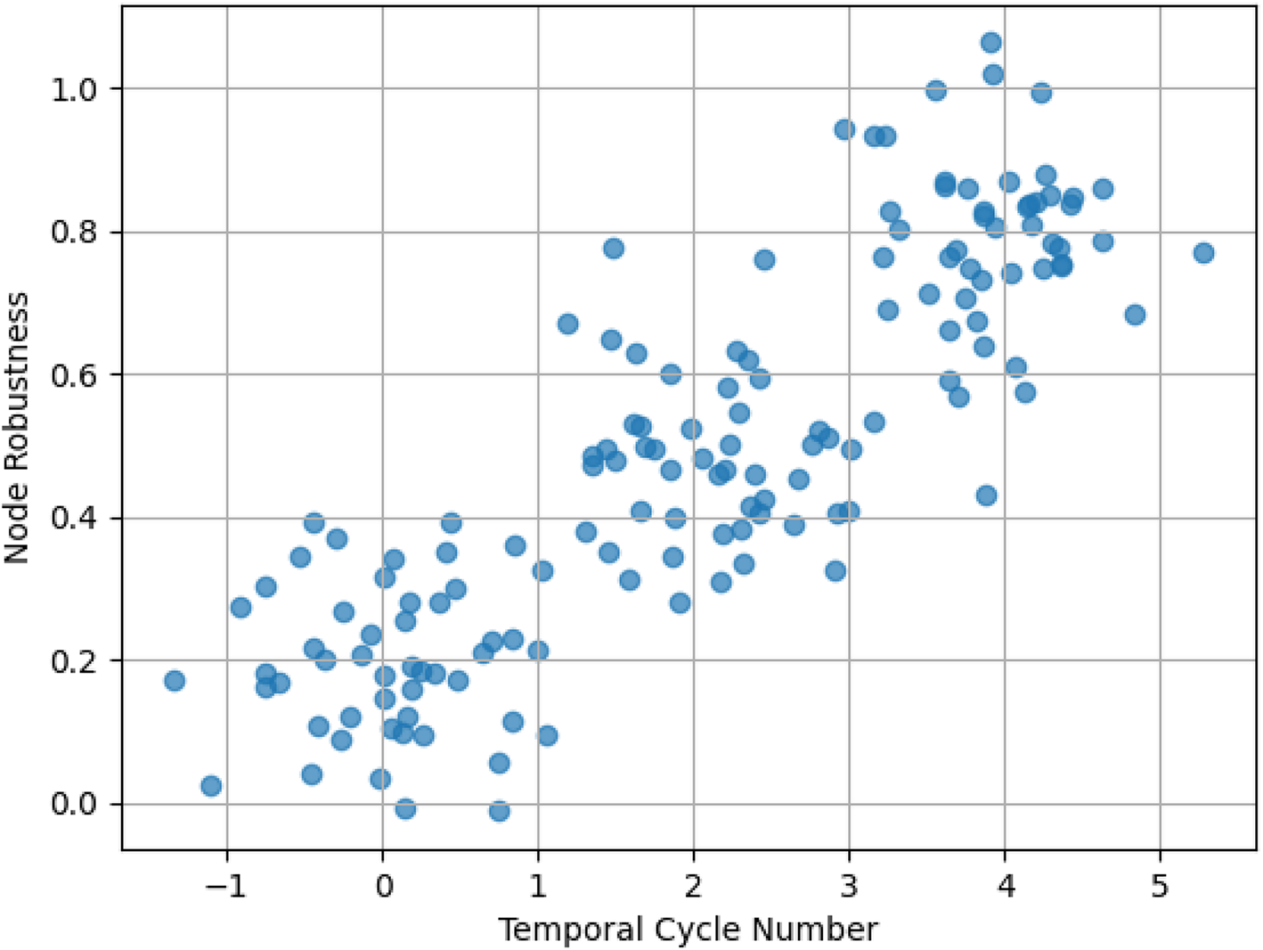
Node resilience profiles across different temporal cycle participation levels.

To interpret how cycle participation stratifies nodes, Fig. 4 relates temporal cycle number to node robustness under increasing disruption intensity.

Nodes with low TCN tend to experience rapid efficiency deterioration once local redundancy is removed, whereas nodes with high TCN remain comparatively stable until disruptions become severe. This pattern supports the “cycle anchor” interpretation: nodes embedded in persistent cycles contribute disproportionately to time-respecting alternative routes. Importantly, high TCN nodes are not necessarily the highest-degree nodes, indicating that cycle-based redundancy is not reducible to raw activity. In other words, the proposed metrics distinguish structural “backbone” participation from mere interaction frequency.

### Temporal evolution case study: transportation network

**Fig. 5:**
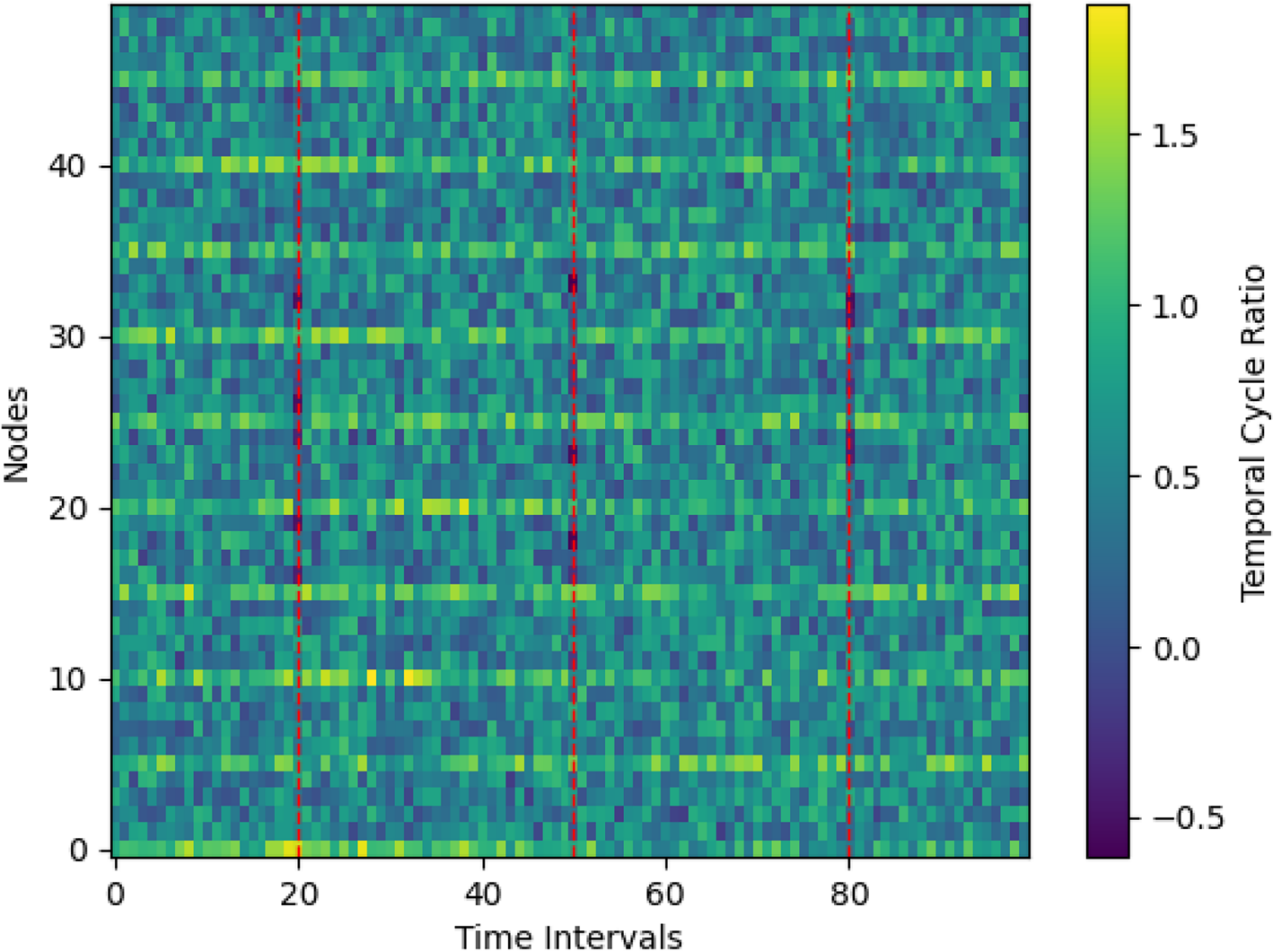
Evolution of cycle participation patterns across network nodes and time intervals.

Figure 5 visualizes temporal cycle ratios across nodes and time intervals for the transportation network.

Several patterns emerge. First, a subset of nodes maintains persistently high TCR over long periods, consistent with stable redundancy backbones. Second, periodic TCR surges coincide with regular operation phases, indicating that cycles can be schedule-driven rather than purely random. Third, disruption intervals align with localized drops in nearby nodes’ TCR, suggesting that cycle participation is sensitive to structural perturbations. Together, these observations support a practical reading of TCR as a monitorable signal: it highlights where durable, time-ordered alternatives concentrate and when they weaken.

### Robustness under disruption strategies

We compare three disruption strategies: random failures(RF), degree-based attacks(DBA), and cycle-based attacks(CBA) guided by TCN/TCR ranking. Table 6 summarizes the resulting efficiency losses.

**Table 6:** Network robustness under different disruption strategies.

Strategy	Social $\Delta E$	Transport $\Delta E$	Biological $\Delta E$
Random failures	0.32 ± 0.04	0.28 ± 0.03	0.25 ± 0.02
Degree-based attacks	0.51 ± 0.05	0.46 ± 0.04	0.39 ± 0.03
Cycle-based attacks	0.63 ± 0.06	0.59 ± 0.05	0.54 ± 0.04

Importantly, the advantage of TCN/TCR-guided removals is not specific to temporal efficiency: consistent separations are also observed on reachability loss and temporal fragmentation (Table 7), indicating that the gains reflect robust detour removal and connectivity breakdown rather than a single chosen metric.

**Table 7:** Multi-metric robustness comparison under different disruption strategies.

	Social			Transport			Biological		
Strategy	$\Delta E$	$\Delta \textrm{TRR}$	$\Delta \textrm{LTCC}$	$\Delta E$	$\Delta \textrm{TRR}$	$\Delta \textrm{LTCC}$	$\Delta E$	$\Delta \textrm{TRR}$	$\Delta \textrm{LTCC}$
RF	0.32	0.18	0.12	0.28	0.21	0.15	0.25	0.16	0.10
DBA	0.51	0.36	0.29	0.46	0.40	0.33	0.39	0.30	0.24
CBA	0.63	0.49	0.41	0.59	0.52	0.45	0.54	0.42	0.36

Cycle-guided removals induce larger efficiency losses than degree-based attacks and random failures under the same removal budget. Across repeated trials, paired comparisons indicate that the differences are statistically significant (typically $p<0.01$), supporting the view that persistent cycles encode vulnerability not captured by degree prominence alone. This result is consistent with the “cycle anchor” mechanism: targeting nodes that hold durable closure removes time-respecting detours more effectively than targeting high-activity nodes.

### Hypernetwork-level properties of persistent cycles

The temporal hypernetwork representation enables compact accounting of persistent cycles. Across datasets, only a minority of detected cycles exceed $\tau _{min}$, yet these persistent cycles account for a large share of total node-level cycle participation. The resulting hypernetwork exhibits hub-like heterogeneity in hyperedge participation, implying that a small set of persistent cycles concentrates redundancy and may form higher-order “backbones” for resilience. Rather than asserting a universal distributional law, we interpret this pattern as evidence of strong concentration, which is consistent with the observed vulnerability of cycle anchors under targeted removals. From a modeling perspective, this concentration justifies encoding persistent cycles explicitly: it yields a compact representation that preserves the structures most responsible for resilience differences.

### Empirical comparison with alternative temporal motif baselines

To complement the conceptual justification for focusing on cycles, we directly compare TCN/TCR against analogously constructed scores based on *persistent 2-paths* (open triads), the most prevalent non-closed temporal motif. Both motif families are processed through the identical persistence-tracking pipeline with the same $\tau _{min}$ selection rule, ensuring that differences in predictive performance reflect the *structural property of closure* rather than pipeline asymmetries.

Table 8 reports rank-based association with disruption impact $\Delta E$ for each motif-derived score across six datasets. Cycle-based TCN/TCR consistently yield higher associations than the 2-path-based counterpart (denoted $\textrm{P2N}$/$\textrm{P2R}$), with an average gap of approximately $+0.08$–$+0.12$ in Spearman’s $\rho$.

The advantage is most pronounced in the transportation and infrastructure networks, where strict temporal ordering leaves fewer feasible paths and closed-loop redundancy becomes the primary alternative-route mechanism. In the social network, where interaction density is higher, the gap narrows but cycles remain modestly superior.

These results provide the empirical support requested: beyond the theoretical argument that cycles are the minimal closed redundancy unit, the data confirm that persistent *closed* motifs carry more disruption-relevant information than persistent *open* motifs of comparable order under the adopted evaluation protocol.

**Table 8:** Empirical comparison of cycle-based vs. 2-path-based resilience scores (Spearman’s $\rho$ with disruption impact $\Delta E$). P2N/P2R = persistent-2-path analogues of TCN/TCR, computed under the identical persistence-tracking pipeline; P2R is normalized by the same cumulative degree denominator as TCR (Eq. (8)).

Metric	Social	Transport	Biological	Communication	Infrastructure	Economic
TCN (cycles)	0.87	0.82	0.91	0.85	0.88	0.83
TCR (cycles)	0.85	0.79	0.89	0.82	0.86	0.81
P2N (2-paths)	0.78	0.69	0.81	0.74	0.72	0.74
P2R (2-paths)	0.76	0.67	0.79	0.72	0.70	0.72
T-Bet (baseline)	0.72	0.68	0.75	0.71	0.69	0.70

All motif scores are computed using the identical persistence-tracking pipeline and $\tau _{min}$ selection rule. Differences in $\rho$ reflect motif type (closed vs. open) rather than pipeline asymmetry

## Discussion

### Interpretation: what persistent cycles capture

Our results suggest that persistence is an informative feature that can make cycle closure more actionable as a redundancy signal under time ordering. Snapshot-based cycle statistics can be inflated by short-lived edges that never co-occur within the adopted temporal aggregation; in contrast, cycles that recur across windows are more likely to coincide with repeatedly available time-respecting alternatives, and therefore can be more aligned with resilience outcomes in the disruption experiments considered here.

This interpretation also offers a plausible explanation for why TCN/TCR may outperform degree- or path-prominence indicators in strongly time-ordered settings *within our evaluation*. High-degree nodes may be active without repeatedly participating in closed temporal structures, and shortest-path prominence does not necessarily reflect the availability of detours when time ordering is enforced. By focusing on recurring closure, the proposed metrics highlight a set of “cycle anchors” whose removal is associated with larger temporal-efficiency loss in our experiments.

The motif comparison in “Empirical comparison with alternative temporal motif baselines” reinforces this interpretation at the empirical level. Persistent 2-paths, despite being processed through the same persistence pipeline, yield lower rank-based associations with disruption impact than cycle-based scores. This gap is consistent with the theoretical distinction: an open triad guarantees a two-hop connection but not a *closed* alternative route, so its removal need not eliminate a detour in the same direct sense as removing a cycle anchor. The empirical advantage of cycles over 2-paths therefore is not merely an artifact of the framework architecture, but reflects the structural property of *closure* as a resilience-relevant feature under time-respecting constraints.

### Implications and potential applications

The results suggest practical value for cycle-aware monitoring and intervention. In infrastructure networks, identifying cycle anchors can guide targeted reinforcement or redundancy planning^42^. In biological systems, persistent cycles may indicate robust regulatory or neural motifs that buffer functional disruption^43^.

Methodologically, the findings encourage shifting part of resilience analysis from purely path-centric indicators to higher-order temporal redundancy structures. The temporal hypernetwork layer also provides a reusable encoding idea for other higher-order temporal patterns, such as persistent cliques or multi-node feedback motifs^44^.

Conceptually, the proposed method can be generalized by replacing the cycle detector with a motif detector *M* that extracts instances of a target temporal pattern (e.g., persistent *k*-cliques, persistent 2-paths, or persistent feed-forward/feedback motifs). The persistence indicator $\phi (\cdot ,t)$ and duration $\tau (\cdot )$ can be defined *identically* for motif instances, and the hypernetwork layer simply encodes each retained motif instance as a hyperedge over its participating node set. Node-level scores then follow the same construction as Eqs. (7)–(8) by counting (or weighting) motif participation rather than cycle participation. In this paper we focus on cycles because they are the lowest-order closed motifs that directly operationalize detours, but the pipeline is designed to support broader motif families with minimal changes.

In this sense, persistent cycles serve both as an interpretable mechanism (redundant detours) and as a compact higher-order representation for analysis and ranking.

### Limitations

While the proposed metrics perform well across heterogeneous temporal networks, several limitations remain.

First, temporal cycle detection can be computationally demanding as network size and duration increase. Although optimizations make the framework practical for medium-scale datasets, large-scale deployments may require approximate counting, sampling, or incremental updates that avoid full recomputation when windows slide.

Second, the current formulation weights cycles primarily by persistence duration and assigns equal within-cycle contribution to all participating nodes in the main analysis. Different cycle topologies (e.g., length, chord structure, temporal spacing) may contribute unequally to effective redundancy, and treating them uniformly can blur fine-grained resilience mechanisms. Incorporating topology-aware weights or length-regularized contributions is a natural extension.

Third, the hypernetwork threshold $\tau _{min}$ requires calibration. Overly strict thresholds may discard informative recurring but intermittent cycles, while lenient thresholds may include noisy transient loops. Although our cross-validation procedure provides a principled selection rule, domain-specific constraints (e.g., operational schedules or biological rhythms) may motivate adaptive thresholding tied to exogenous time scales.

Finally, the framework interprets persistent cycles as beneficial redundancy, which holds for many infrastructure and communication settings. In social or information networks, however, persistent closed loops can also reinforce undesirable dynamics (e.g., echo-like reinforcement), meaning that “resilience” may need to be defined relative to the target function rather than connectivity alone. Extending the approach to function-specific notions of resilience is therefore important for applications where connectivity is not the sole objective.

### Future work

Several extensions are promising. First, predictive modeling of cycle formation and dissolution could support proactive resilience management, where interventions are triggered before redundancy backbones degrade. Second, multiplex settings may require cycle definitions that span interaction layers^45^, where cross-layer loop persistence drives robustness. Third, real-time monitoring motivates incremental cycle tracking algorithms that update cycle inventories efficiently under streaming edges^46^.

Finally, formal analysis linking cycle statistics to resilience bounds under different disruption models would complement our empirical evidence and clarify when persistent cycles are necessary versus merely correlated with robustness. Such theory would also help translate cycle-based rankings into decision rules under explicit operational constraints.

## Conclusion

This paper proposes a cycle-driven resilience framework for temporal networks that explicitly models persistent cyclical structures as higher-order units. By combining temporal cycle tracking, a temporal hypernetwork encoding of persistent cycles, and two dynamic node-level metrics (TCN and TCR), the approach captures structural signals that can be obscured by snapshot aggregation or purely path-based indicators.

Across six real-world temporal networks, our experiments show that persistence-weighted cycle participation is associated with disruption impact under the adopted windowing scheme and node-removal protocols. Nodes strongly embedded in persistent cycles can act as “cycle anchors” in the sense that their removal is *often* accompanied by larger temporal-efficiency degradation in the controlled tests reported here. The hypernetwork layer further suggests that persistent-cycle participation can be concentrated in a subset of retained cycles, offering a compact representation for higher-order temporal structure aligned with the evaluated resilience measures.

Beyond empirical performance, the main contribution is a persistence-aware view of redundancy: cycles are most informative for temporal resilience analysis when they recur under the adopted temporal aggregation, rather than being observed only in an aggregated snapshot. This perspective complements existing temporal-path and centrality-based approaches by providing an interpretable structural signal and a practical ranking criterion for disruption analysis.

Additional motif-substituted comparisons suggest that closed recurring motifs are more closely aligned with detour-preserving resilience than representative open temporal motifs under the evaluated settings, further supporting the use of persistent cycles as the main structural signal in the present framework.

Future work can improve scalability via approximate or incremental cycle tracking, refine topology-aware cycle weighting, and extend the framework to multiplex networks and function-specific notions of resilience. Overall, the proposed cycle-driven pipeline advances temporal network resilience analysis by shifting attention from edge- and path-centric summaries to interpretable higher-order temporal structures that sustain robustness in dynamic systems.

## Data Availability

All datasets analyzed in this study are publicly available from their original providers. The dataset name, primary source/platform, and direct access link are provided below: Social Interactions (face-to-face contacts). *Hypertext 2009 face-to-face proximity network* provided by the *SocioPatterns* project (wearable sensor/RFID proximity data collected at ACM Hypertext’09). Direct access: https://sociopatterns.org/datasets/hypertext-2009-dynamic-contact-network/. Transportation Systems (air traffic). *OpenSky Network flight data* provided by the *OpenSky Network Association* (crowd-sourced air traffic surveillance data and APIs for research use). Direct access: https://opensky-network.org/. Biological Networks (neural recordings). *hc-3: Multiple single unit recordings from rat hippocampal and entorhinal regions* hosted by *CRCNS.org* and contributed by the *Buzsáki Lab (New York University)*. Direct access: https://crcns.org/data-sets/hc/hc-3. (Note: access may require free registration and acceptance of CRCNS data-use terms.) Communication Networks (email). *Enron Email Dataset* archived and distributed by *Carnegie Mellon University*. Direct access: https://www.cs.cmu.edu/~./enron/. Infrastructure Networks (power grid). *US power grid network data* (commonly used Western States power grid topology) curated by *Mark E. J. Newman* as part of the network data repository. Direct access: http://www-personal.umich.edu/~mejn/netdata/. Economic Networks (international trade). *UN Comtrade* international trade statistics provided by the *United Nations Statistics Division*. Direct access: https://comtrade.un.org/; API portal: https://comtradedeveloper.un.org/. Monthly trade flows used to construct temporal trade networks can be retrieved via the UN Comtrade API (e.g., a query template such as https://comtrade.un.org/api/get?max=50000&type=C&freq=M&px=HS&ps=202001&r=156&p=0&rg=all&cc=TOTAL). No new raw datasets were generated in this study. All data preprocessing scripts and derived data products (e.g., windowed temporal snapshots, persistent-cycle inventories/hyperedge lists, and node-level TCN/TCR scores) are available from the corresponding author upon reasonable request.
